# Diversifying relationships: Russian policy toward GCC

**DOI:** 10.1057/s41311-021-00286-4

**Published:** 2021-02-27

**Authors:** Leonid Issaev, Nikolay Kozhanov

**Affiliations:** 1 HSE University, Moscow, Russian Federation; 2 Gulf Studies Center, Qatar University, Doha, Qatar

**Keywords:** Russian foreign policy, GCC, OPEC+, Conflicts, Bargaining strategy

## Abstract

The interaction between Russia and Gulf countries represents the story of ups and downs, severe conflicts and sharp warmings that can largely be explained by the permanently changing role and place of each of these players at the global and Middle Eastern political arenas. After Russia's “return” to the Middle East in 2012–2015, Moscow's foreign policy towards the Gulf can be explained in terms of a bargaining strategy. On the one hand, Russia is trying to underline its importance and relevance to the GCC by putting forward diplomatic and political initiatives. The Kremlin uses its direct or indirect presence in the key regional conflicts such as the Syrian, Libyan and Yemeni civil wars as well as the Israeli-Palestinian conflict and Iran’s nuclear issue. On the other hand, Russia is interested in building up stronger economic cooperation with the GCC, drawing bigger volumes of investments from the Gulf to Russia’s broken economy, as well as coordinating efforts with Saudi Arabia in the global oil market. While, in the near future, the qualitative evolution of Russia’s relations with the GCC is hardly possible, there are still options for their deepening within the current level of interaction between Moscow and the Gulf.

## Introduction

During the second part of 2010s, Russian relations with the GCC have developed on a largely positive trajectory. Even Moscow’s decision to leave the OPEC + agreement and launch a price war with Saudi Arabia in March 2020 did not threaten the gains made by Russian diplomacy in the region. Following a cooling-off period in 2012–2014 caused by the negative reaction of the GCC States to the Russian support of the Assad regime in Syria, the overall rapprochement continued based on a complex mixture of factors that include the growing intensity of Moscow’s presence in the region, changing dynamics of the US relations with Russia and the GCC, the evolution within energy markets, existing tensions between Middle Eastern countries, as well as the transformation of GCC foreign policy vision. This paper will look into the influence of these factors on the current development of Russia’s relations with the GCC including an analysis of the ability of the sides to bring these relations to a new qualitative level.

Earlier, we have already shown that Russian policy towards the countries of the Middle East and, in particular, the Gulf countries is based on two pillars: balancing policy and bargaining strategy (see, e.g. Issaev and Korotayev 2020; Kozhanov 2018, 2020; Kozhanov and Issaev 2017). In other words, pragmatism is the determining factor in Russia's cooperation with any country in the region. Its policy is free of messianism, and Russia cannot be viewed as part of any kind of alliance. At the same time, the Russian presence in the Syrian and Libyan conflicts, as well as indirect influence on the Yemeni and other crises in the Middle East through the veto in the UN Security Council, gives the Russian leadership the opportunity to exchange the military-political advantages it gained for economic projects with the countries of the region and primarily the Gulf.

## Historical background

During the peak of Soviet influence, the Gulf was an arena of Soviet-American confrontation. After the Soviet Union’s collapse, Russia became less active in the region. With some countries of the Gulf (UAE, Kuwait, Oman), the Soviet Union and then Russia developed relatively stable relations. For Moscow, the strategic cooperation between the Gulf monarchies and the USA was obvious. At the same time, the main driver of bilateral cooperation was not so much geopolitical motives, but a pragmatic interest in the development of certain political and economic projects. This can be illustrated by the past record of Russia’s relations with Saudi Arabia and Qatar.

Thus, the Soviet Union was the first country to recognize the independence of the Saudi state created by King Abd al-Aziz in 1926. However, this did not prevent the Saudi leadership from breaking diplomatic relations with the USSR 12 years later after the Soviet ambassador Karim Khakimov was recalled to Moscow and shot on charges of espionage as part of the anti-religious campaign that gained momentum (Barmin 2017). Relations between Russia and Saudi Arabia were restored only in 1990. The joint Russian-Saudi communique stressed that both countries sought to develop “friendly relations for the benefit of the peoples of both states,” promoting “the settlement of regional conflicts, the development of international cooperation and the strengthening of peace and international security” (Izvestiya 1990). Despite this, a lot of disagreement remained between Russia and Saudi Arabia in the 1990s, especially on political issues. One of the most acute, of course, was the situation in the North Caucasus.

The subsequent history of Russian-Saudi relations largely depended on the state of relations between Riyadh and its main geopolitical ally, the USA. After the terrorist attacks in New York on September 11, 2001, the US-Saudi cooperation deteriorated sharply. The USA claimed that Saudi Arabia had become a dangerous opponent of the USA. Under these conditions, it became possible to symbolically improve Russian-Saudi relations.

The next stage of rapprochement between Saudi Arabia and Russia was also connected with the invasion of the USA to Iraq in 2003. Moscow argued against the actions of the USA, and the voice of the Kingdom which refused to participate in the anti-Iraqi coalition, was not conceived by Washington. At the same time, Saudi criticism of Russia's policy in the North Caucasus was muffled. In January 2004, Saudi Arabia accepted the Chechen leader Akhmad Kadyrov as a legitimate representative of the Chechen people. Moreover, he received a high honor: along with Prince Abdullah he participated in the Washing of the Kaaba (Vasiliev 2016: 9). And in 2008, after the conflict in South Ossetia, King Abdullah said that he “understands Russia's actions” (Vasiliev 2016). However, this was not followed by the recognition of the independence of South Ossetia and Abkhazia by the Kingdom.

Thus, it can be said that the 2000s became a period when both countries sought to bring together, but the fragility of this process was largely provoked by external circumstances, rather than by the internal needs of the two countries. As a result, the remaining disagreements on many issues, lack of trust and negative image against each other formed by decades [anti-Russian materials in the Saudi media were published with the same regular consistency as the accusations of the Kingdom by Russian media in its adherence to medieval customs and complicity in world terrorism (see, e.g., Kosach et al. 2017)] led to the fact that the desire for cooperation was preserved only on paper. The trade between Russia and Saudi Arabia, which by 2009 amounted to only $363.8 million, is the brightest indicator of this (Embassy of Russian Federation to Saudi Arabia 2010).

Since the fall of 2001, Saudi Arabia and other OPEC member states (such as Kuwait, the UAE and Qatar) have unsuccessfully tried to get Russia to reduce oil production in order to keep prices high. Moscow refused, but prices soon rose without it, putting oil disputes on the back burner. In 2008, Russian Railways won a tender for the construction of a 520-km railway line worth $ 800 million, but four months later Saudi Arabia refused to sign the contract. This purely political decision of the Saudi leadership, on the one hand, demonstrated the real attitude of the Kingdom to the expansion of cooperation with Moscow and, on the other, coincided with the next period of normalization of relations with Washington. In 2008, when Barack Obama became the US president, Riyadh had high reliance upon him. Suffice it to recall his first visit to Saudi Arabia, with which he began his first tour of the Middle East, culminating in his speech at the University of Cairo, where the American president addressed the entire Muslim community with his vision of the coming American Middle East policy. Under these conditions, Russia once again ceased to represent any interest for Saudi Arabia, which predetermined another period of stagnation in the relations between the countries.

Additionally, during the 1990s–early 2000s, Russia suspected that private individuals from Saudi Arabia and other GCC countries in funding Islamist radical groups in the North Caucasus (Yurchenko 2003). From 1997 to 1999, according to a representative of the US state Department, charitable organizations from the Gulf countries allocated more than $100 million to support Chechen separatists (see, e.g., Vasiliev 2016, 2018). Statements by the Saudi officials were occasionally no less discouraging. However, at the government level, Saudi Arabia and other GCC member states showed restraint and declared their unwillingness to interfere in Russia's internal affairs (see, e.g., Kosach 2005).

Diplomatic relations between the Soviet Union and Qatar were established quite late, only in 1988, 17 years after this Arabian monarchy declared its independence. After the collapse of the Soviet Union, Doha had good relations with Moscow. In April 1998, Qatar and Russia even signed a military cooperation agreement, which, however, was not implemented. Qatar's foreign Minister, Sheikh Hamad bin Jasim bin Jaber al-Sani, was visiting Moscow at the time, and his talks with foreign Minister Yevgeny Primakov were described as “very successful” (Vasiliev 2016). However, as with many other countries in the region, it did not go beyond words.

The September 11, 2001, attacks soured US relations with the Arabian monarchies, which opened up new opportunities for Moscow to develop ties with Doha. In addition, at this time, the Chechen conflict also began to decline. Russia sought to get closer to the Muslim world and even began to participate in the work of the Organization of the Islamic Conference (now the Organization of Islamic cooperation). In 2003, Vladimir Putin first attended the OIC summit, which was chaired by Qatari Emir Hamad bin Khalifa al-Thani. But hopes of deepening cooperation collapsed in 2004, when one of the Chechen separatist leaders, Zelimkhan Yandarbiyev, was killed in Doha by the Russian security forces. Yandarbiyev was considered a personal guest of the Emir of Qatar, and therefore his elimination, which became the most high-profile crime in the history of the Emirate, was perceived by Hamad al-Thani as a personal insult.

The situation with Yandarbiev has severely damaged the bilateral relations. They further worthened after November 29, 2011, when Ambassador Vladimir Titorenko, who arrived in Doha with the diplomatic post, was beaten at the capital's airport while Qatari customs officers tried to search him. Russia had to call its Ambassador back from Doha and lower the level of diplomatic relations with Qatar. The situation improved only in 2013 after the abdication of Hamad al-Thani in favor of his son Tamim. His rise to power positively boosted not only the political dialogues between the two countries but also their trade and economic relations.

## Russian priorities in the Middle East

Russia’s growing involvement in the Middle East over the past years has brought about plethora of authors looking into different aspects of its regional presence. In these studies, scholars have also begun to address in more detail the issue of Moscow’s relations with the GCC (see, e.g., Borshchevskaya 2016; Katz 2018a, b; Ramani 2020a; Shumilin and Shumilin 2017; Sim 2018). At the same time, the majority of recently published studies on the Russian presence in the Middle East still only touch upon Russia’s ties with the GCC members states within the broader discussion of Moscow’s regional approaches thus not extending sufficient attention (see, e.g., Rumer 2019). Those that do not tend to be too narrow are either concentrating on the dynamics underlying bilateral Russian-GCC ties with a selected GCC country or focusing on just one aspect of the Russia’s dialogue with the GCC, primarily the energy dimension (Bradshaw et al 2019).

Mark Katz provides a comparison of the strengths and weaknesses of Soviet policy toward the Gulf during the Cold War with Russia’s vision of the region after Putin’s rise to power in 1999. The idea of the continuity of Moscow’s policies towards the region is often ignored by other scholars who instead begin their story of Russia’s relations with the Gulf from the early 2000s. This largely ignores Moscow’s ties with the region during the 1990s and the fact that it represented interest for the Soviet strategists who believed that the political and military presence of the USSR in the Gulf can strengthen its positions in the Cold War (Vasiliev 2018). Katz, on the contrary, examines both Soviet and Putin’s foreign policies and explores both what went well for Moscow and what did not, thus, offering an explanation of the Soviet gains and misfortunes in the Middle East. Still, even here the discourse on Russian relation with the GCC is overshadowed by the analysis of Russian connections with other regional players such as Iraq, Iran and Yemen (Katz 2018a).

What Katz correctly demonstrates is that while Moscow’s decisions obviously play a role in whether its Middle Eastern policies have been successful or not, the determination of Moscow’s successes or failures is as much a result of Moscow’s own decisions as it is the result of policies pursued by the US, Middle Eastern or other actors (Katz 2018a). The analysis of these ties through the prism of Russia’s relations with the West and its ambitions to play the role of a global player at the international arena has, in general, become a very popular way to approach the issue (Ramani 2019). Such approach, however, fails to adequately account for important domestic and economic factors that are at least equally as significant (see, e.g., Issaev and Shishkina 2020).

Russia’s dialogue with Saudi Arabia on the OPEC + for example is often considered within the framework of Moscow’s vision of the global energy markets rather than from the point of its meaning for the Russian dialogue with the region itself (see Bradshaw et al. 2019; Sim 2019).

At the same time, Russia’s foreign policy towards the Gulf is inseparable from its broader diplomacy in the Middle East. Since the beginning of Russia’s military deployment in Syria in 2015, Moscow importance for regional and non-regional players has been based on two pillars: an expanding Russian military presence in the Middle East (first of all, in Syria, but also in Libya) and a “pragmatic” balancing between key regional players (see, e.g., Rumer 2017; Donaldson and Nadkarni 2018; Berthelot 2017). That core set of priorities shaping Moscow’s approaches to the region, and consequently the GCC has remained unchanged. This list includes Russia’s ambitions to project power, the Kremlin’s endless quest for economic profit and Moscow’s domestic concerns. What has shifted over the last five years is the nature of the impact of these factors on Russian strategic thinking. These factors are constantly evolving in turn adding new shades to the Kremlin’s approaches. Specifically, since 2015, the influence of economic considerations on Russia’s regional decision-making has become much more obvious.

In the geopolitical sphere, Russia’s leadership always looks at the Middle East and the Gulf through the lenses of its goal of projecting power globally and confronting the West (see, e.g., Trenin 2017; Vasiliev 2018; Vasiliev et al 2019). Regional priorities play a secondary role. Moscow’s involvement in the conflicts in Syria and Libya, its close contacts with the Palestinian authorities and Israel, as well as attempts to maintain good ties with Iran, on the one hand, and the GCC, on the other, seek to underline to demonstrate to the US and the EU Russia’s importance as a global player, thus compelling the West to further take Russia’s worldview into account and to keep communication channels with Moscow open.

Russia’s policy initiatives in the Gulf region here are illustrative. In July 2019, Russia released its “Concept of Collective Security in the Persian Gulf” believing that current tensions in the region would mean the international community will be ready to support such initiative. The Kremlin further believes that it will be able to use this document not only in the Gulf, but further afield too—again to show that Russia plays an important international role. Russia’s diplomats The Ministry of Foreign Affairs also argues that the publication of the “concept” is a response to attempts by Western actors to impose a “western solution” on the region. Ultimately, the Kremlin believes that the emergence of a new security system in the Gulf is inevitable. Yet, given its ambitions to play a larger role in this part of the region and beyond, it does not want this new system to be established without its participation. There are few expectations in Moscow that the concept will be adopted by others as it is. However, it is intended to secure its position at the table in any discussion on the future structure of international relations of the Gulf.

In order to use the Middle Eastern issues to shape its relations with the West, the Russian leadership has to maintain Russia’s position as an influential external player in the region, including the Gulf. This means that Moscow also needs to demonstrate its importance to the regional players. Under these circumstances, Moscow’s active attempts to maintain good ties with the warring sides in Yemen, its support provided to General Khalifa Haftar in Libya, active cooperation with Saudi Arabia and the UAE in the OPEC + and readiness to open Syria for the economic presence of the GCC countries are, among all, aimed to make the Gulf states take the Kremlin’s worldview into account and to keep communication channels with Moscow open. Moreover, Moscow is not confident that Russia would be able to respond effectively if forced into a reactive mode by other players in the region. The Kremlin therefore seeks to retain the initiative and thus shape the regional agenda according to its needs and resources.

## Russia’s Quid-pro-quo approach

Since the beginning of the operation of the Russian Aerospace Forces in Syria in 2015, the Kremlin has managed to form a stable perception of Russia as an important geopolitical actor in the Middle East, without taking into account the opinion of which the resolution of regional conflicts looks at least difficult. There is no conflict in the Middle East that would be of vital interest to Russia, which makes its policy very flexible. Moreover, Moscow is very willing to sell its “liquid assets.” This, in turn, made it possible for Moscow to adopt a bargaining strategy, and also to offer its services to the countries of the region as an intermediary. This primarily applies to the Gulf countries, which have their own vital interests in Yemen, Libya and Syria. In turn, for Moscow, the possibility of building up investment and energy cooperation with the Gulf region, which is very attractive for Russia under the sanctions, looks extremely attractive.

In Syria, Russia managed to build up in the eyes of the regional players the image of a country willing to engage and be decisive in its actions. This image also emerged not without the exploitation of the US mistakes. The failure by the US to undertake military operations against the Assad regime in Syria in 2013 followed by Russia’s own military deployment in the country after 2015—was vital for strengthening the perception of Moscow as an influential player in the eyes of the GCC. Russia’s military deployment in Syria became not only the symbol of the Russian decisiveness to protect its allies but clearly showed that Russia’s involvement could in fact be a game changer. It thus contrasted sharply with the US approach to the Mubarak regime in Egypt. Moreover, the military operation ensured not only the survival of the Assad regime but further demonstrated that the Western monopoly on the use of force in the Middle East that emerged after the fall of the USSR was over. For the GCC, this was an acknowledged that Russia should no longer be considered a weak player when comes to the larger strategic picture in the region.

Initially, Russian support for Bashar Assad remained one of the main deterrents for the development of the Russian contacts with the GCC states. From the outset of the civil war, Riyadh heavily criticized the Kremlin for its stance on the conflict (Browning and Maclean 2015). Yet, in order to shape the situation in Syria in its interests, Russia understood that it also need to open channels of communication with Saudi Arabia, the UAE and Qatar. Through the deployment of its military forces, Moscow put pressure on those groups that were receiving support from Saudi Arabia. As the pressure on those groups increased, Saudi Arabia felt the need to talk to Russia. This, in turn, led Moscow to offer a number of incentives for the intensification of the political dialogue on Syria. By mid-2017, for example, Russia supported Saudi efforts to assemble Syrian opposition group with the idea to put together one unified opposition that could take part in talks of a peace settlement taking place in Geneva. The Kremlin also demonstrated to Riyadh that there were other topics of mutual interest (including the situation in the international oil market) that could be discussed if the disagreements on Syria were either put aside or overcome.

Also, the Kremlin underscored its readiness to give certain concessions to Saudi Arabia in exchange for the reciprocal moves of Riyadh. Again, by mid-2017, Russia agreed not to voice objections against Saudi actions in Yemen in exchange for a more flexible position on Syria by the Kingdom. Subsequently, on January 21–23, 2018, Russia hosted the visit of Abulmalik al-Mekhlafi, the foreign minister of the Saudi-supported Yemeni government of Abdrabbuh Hadi. Previously, Moscow had supported Hadi’s opponents from former Yemeni President Abdullah Saleh’s team. As a formal gesture of support from the Russian side, Moscow agreed on a formal request by the Hadi’s government to send 50 tons of grain to Yemen (Belenkaya 2018).

After the Coalition led by Saudi Arabia launched the military operation “Decisive Storm” on March 25, 2015, Russia once again supported—first de facto and then de jure—the actions of Riyadh (see, e.g., Blumi 2017; Issaev and Korotayev 2015). It is noteworthy that, in contrast to its categorical assessment of the actions of the USA and NATO in relation to Yugoslavia, Iraq, Libya and Syria, the Russian authorities tried to avoid direct criticism of the actions of Saudi Arabia in Yemen, despite the absence of sanction from the UN Security Council.

Russia tried to avoid a legal assessment of the actions of the Arab Coalition in Yemen, limiting itself to calls for an early cessation of hostilities, as well as blaming the escalation of the conflict not only on the GCC, but also on their opponents in Yemen in the person of the Houthis and the People’s General Congress. Notable here is the April 14, 2015, vote on Resolution 2216 in the UN Security Council. This resolution did not mention the actions of the Arab Coalition, instead blaming the situation primarily on the Houthis (UN SC 2015). The Russian delegation abstained from voting, which was tantamount to supporting the actions of Riyadh, and gave the green light to the Saudi leadership in its actions in Yemen.

However, already in February 2016 and 2017, the Russian delegation supported the vote in favour of the new Resolutions on Yemen proposed by the UK—2266 (UN SC 2016) and 2342 (UN SC 2017). Nevertheless, for Russia Yemeni issue had moved to the periphery of Russian politics in the Middle East. By 2016, the OPEC + format, which became one of the central issues in Russia's relations with the Gulf countries, was formed.

Libya might be another example of occasional interaction between Russia and some of the GCC members (first of all, the UAE) based on the principles of pragmatism and overlapping interests without the formation of a long-term coalitions. Thus, there are occasional attempts by some analysts to position Moscow and Abu Dhabi as partners in Libya united by the necessity to provide support to General Khalifa Haftar (Ramani 2020b). However, there are certain difficulties with finding proofs for such assumptions. Until the early spring 2020, there were little evidences of viable policy coordination between Russia and the UAE on Libya apart from certain speculations on the Emirates’ financial support allocated to General Haftar in order to pay for Russian military supplies and services of Russian mercenaries from PMC Wagner (allegedly owned by the Russian tycoon and member of Putin’s inner circle, Yevgeny Prigozhin) (The New Arab 2020). However, even putting aside questions regarding the credibility of other facts mentioned in these speculations (such as, for instance, Russia’s direct arms supplies to Haftar or the scale of Russia’s mercenaries involvement in the conflict), none of them clearly proves that the two countries deliberately agreed to divide the roles in their support of Haftar or somehow coordinated the efforts.

The absence of any viable Russian-UAE alliance can be explained by the fact that while being interested in supporting Tobruk (including Aguila Saleh’s peaceful initiatives) Moscow and Abu Dhabi have different motivations and priorities in Libya. Russia considers its involvement in Libya as an adventure that has nothing to do with the national security or Russia’s core national interests. To a certain extent, Moscow is gambling: it wants to see what it can politically and economically get from its involvement in Libya whose cost for Russia is not high. Russia and the UAE also have different degree of loyalty to Haftar (Ramani 2020b). Moscow whose foreign policy strategy in the Middle East is based on the idea of balancing between all players do not want to join one camp and flirt with Haftar’s opponents as well. Inside the Russian elite there is also no unanimity about Haftar and necessity to see him as the only figure who can secure Russia’s interests in Libya. Moscow also has little trust to him.

Consequently, the intensity of Russian-UAE contacts on Libya that has been growing since the winter of 2020 should not be deceiving: it is another occasional interaction between the two countries whose diplomatic courses on Libya were temporarily brought together by the developments on the ground (Bystrov 2020). Both Russia and the UAE are concerned with Haftar’s military misfortunes experienced by his army during the spring—early summer 2020. The two countries are worried about growing Turkey’s involvement in the conflict and the US joining it on Tripoli’s side. They see the revitalization of political track as the only way to decrease the intensity of clashes and freeze the situation on the fronts. At the same time they want to promote their vision of the conflict settlement spanning around so-called Cairo declaration and Aguila Saleh’s initiatives (Belenkaya 2020). And that’s where Moscow and Aby Dhabi are compatible: while Moscow is using its contacts with Ankara and Paris to come to terms with them about the ideas stated in the Cairo declaration, the UAE, as argued by some analysts, is discussing them with the US. Finally, it is also important that, according to the Russian analysts, Russia and the UAE have the same vision about military “red lines” in Libya. For them, Sirte and al-Jufra air base are to remain in the hands of Tobruk (Bystrov 2020).

Yet, even under the current circumstances, Russia and the UAE show little evidences for forming a full-fledged alliance. Haftar’s problems does not change Moscow’s opportunistic vision of the conflict. As a result, there is no motivation for the Kremlin to change its logic of interaction with those countries who also support Tobruk. Also, the formation of a long-term alliance with the UAE on Libya would inevitably backfired at Russia’s relations with Qatar and make Moscow’s dialogue more complicated than it is (Vasiliev et al 2020).

From a Russian perspective, the quid-pro-quo approach has brought about the necessary results. For one, Saudi Arabia helped Moscow to launch the dialogue with the part of the Syrian opposition supported by Riyadh. Second, in 2018, Saudi Arabia was seen as giving its silent consent regarding the intentions of Bahrain and the UAE to re-open their embassies in Damascus and providing limited economic assistance to the reconstruction of Syria (RIA Novosti 2019). Third, Russia also gradually persuaded Riyadh to support the Syrian return to the League of Arab States and in this context some progress in the discussion over Saudi assistance in the reconstruction of Syria is also reported.

## Friendship with reservations: limits of Quid-pro-quo approach

Yet the quid-pro-quo principle also has its exceptions when it comes to Moscow’s relations with the GCC. For instance, it is absolutely unacceptable for Russia to trade its good relations with other regional actors (primarily with Iran and Israel) for investments and greater political support from the GCC member states. At the same time, Gulf countries are also not ready to abandon strategic relations with the USA and Western countries for the sake of dialogue with Moscow (see, e.g., Al Shayji 2014; Feierstein 2017; Goldenberg and Dalton 2015; Harb 2017; Ulrichsen 2016).

In spite of different attempts undertaken by the GCC countries (first of all, Saudi Arabia and the UAE) to persuade the Kremlin to cool down its relations with Iran, the position of the Russian leadership was clear: these ties cannot be a trading item. Moscow and Tehran collaborate on a wide variety of other regional issues, such as energy and security in the Caspian region and Central Asia. And it is not only Iran who wants these collaborations to continue. Moscow has not forgotten how the civil war in Tajikistan in the mid-1990s was stopped only with effective cooperation with Iran. Similarly, Tehran’s stance during the Russian war with Georgia in 2008 was construed by the Kremlin as de facto pro-Russian. Finally, in 2018, the adoption of a Moscow-backed framework agreement on the legal status of the Caspian Sea would have been considerably more difficult without Iranian consent. In exchange for its diplomatic support, Iran aimed to secure the Kremlin’s further assistance in its struggle against American pressure—although of the five littoral countries that signed the agreement, Iran’s interests were ranked as the lowest priority (Grajewsky 2020).

It is also unlikely that Russia will exert pressure on Iran in Syria to please the GCC. Russia’s ability to confine Iran’s presence in Syria is limited. On the one hand, Moscow still needs Tehran’s proxies on the ground for as long as the war continues, even as it tries to squeeze them out of certain areas. On the other hand, Russia has few effective tools to force Iran, its proxies and/or “pro-Iran forces” to leave Syria. Russia could theoretically advocate for the withdrawal of groups such as Hashd al Shaabi, Afghan and Pakistani fighters and Hezbollah in exchange for concessions to Iran in Syria or elsewhere. Yet there are other local forces supported by Iran such as the National Defense Forces or Local Defense Forces by Syrians, to which Tehran is unlikely to end its support (Mardasov and Semenov 2018).

Consequently, it should by now be clear that any improvement in Russian-Saudi relations will have little noticeable impact on Russia's sometimes prickly but nevertheless longstanding cooperation with Iran. Despite periodic attempts by Saudi Arabia to get Moscow to move towards the anti-Iranian camp, Russian current balancing strategy precludes the likelihood for what it is possible to refer as a “friendship against” a chosen rival. On top of this, Moscow retains its own level of mistrust towards countries such as Saudi Arabia and the UAE while seeing Iran as an occasional partner in its efforts to counterbalance US plans in the region. This in turn makes Riyadh and Abu Dhabi skeptical about Russia’s abilities to put pressure on Iran, for instance, when it comes to decreasing Tehran’s presence in Syria.

The situation around the Arab–Israeli conflict at the turn of the 2010–2020s is also a good example of how Russia and the Gulf countries focus primarily on the bilateral agenda, trying to avoid the discussion of regional issues that can spark tensions between Moscow and the GCC member states. The attempts by Donald Trump’s administration to resolve the Middle East conflict within the framework of the “Century Deal”, as well as the White House's mediation efforts to normalize relations between Israel and the Arab countries (primarily the Gulf countries), caused skeptical and sometimes disapproving comments in Moscow. Speaking at the meeting of the UN Security Council on January 26, 2021, Foreign Minister Sergei Lavrov outlined Russia's position on this issue as follows:

Clearly, the steps to dismantle the international legal framework for a Middle East settlement approved by the Security Council, and replacing collective diplomatic efforts with the “art of the deal” diplomacy cannot produce the desired outcome… Importantly, the process of normalising Israel's relations with the Arab states which was launched in 2020 and which we welcome, should be aimed at stabilising the Middle East region rather than be used to put the Palestinian issue aside, as they say, until better times (Ministry of Foreign Affairs of Russia 2021).

A whole series of normalization of relations between Israel and Arab countries put Russia in a very uncomfortable position. On the one hand, Washington's mediation efforts undermined the already existing mechanisms for resolving the Arab–Israeli conflict (within the UN, the Middle East Quartet, etc.), within which Russia, as a participant, had influence.

On the other hand, due to the fact that the improvement of Israel’s relations with the UAE and Bahrain was driven by the interests of all sides involved Russia was unable to openly criticize the "Abraham Accords" as this would cause condemnation not only in the USA and Jerusalem, but also in Abu Dhabi or Manama. As a result, Moscow has chosen the most universal tactics of appealing to existing international legal norms in order to minimize risks to its own interests. As noted by Alexei Vasiliev, Russia's unnamed position since the collapse of the USSR on maintaining the existing status quo in the Arab–Israeli conflict is largely due to Moscow's fears that this will lead to “the dictates of a stronger side—Israel and its strategic ally, the United States” (Vasiliev 2018).

## Economic motivation with the political aftertaste

First of all, Russia considers the GCC as an important source of investments in Russia’s economy (with the priority of infrastructural projects). The Russian Direct Investment Fund (RDIF) can be considered as one of the main promoters and entry gates for the GCC investments through facilitation of the deals and establishment of joint funds with the Gulf states business and financial entitles. Thus, the list of its partners includes Emirati Mubadala, DP World, Saudi Public Investment Fund, Saudi Aramco, Ayar International Investment Company, Qatar Investment Authority, Kuwait Investment Authority, and Bahraini Mumtalakat. For the last seven years, it brought $2.5 bln of investments from Saudi Arabia, $2 bln of investments from the UAE and less than $1 bln of investments from Kuwait (Shpilevskaya 2019). Qatar investments in Russia’s economy account for $13 bln (Gulf Times 2019). Given that access to the data on investments in Russia is partially restricted, the real volume of money invested in Russia’s economy can be higher. As of 2018, the share of the GCC countries in the RDIF investment funds (including potential projects) was estimated in 52% (Saudi Arabia accounted for 22%, the UAE for 18%, Qatar—8% and Kuwait—2%) (Galaktionova et al. 2018: 17–28).

The strategies of the GCC investors in Russia differ by country. Bahrain and Oman have no known presence in Russia (Bahrain only has a cooperation agreement signed with the RDIF). Kuwait keeps its activities low profile. Thus, in 2012, the Kuwait Investment Authority signed an agreement with the RIDF on the provision of $500 bln (in 2015, this figure was doubled) for future investments in Russia’s economy through so-called automatic co-funding scheme. The scheme implies that Kuwait investor can automatically participate in the RDIF’s projects covering up to 10% of necessary funds. However, there are no confirmed data on any investment’s projects in Russia (Galaktionova et al. 2018). Qatar, the UAE and Saudi Arabia are, on the contrary, much more active. However, their preferred tactics are not the same.

Doha lesser than the others relies on the RDIF in its investment activities and prefers buying shares in large companies such as Russia’s hydrocarbon giant Rosneft, one of the main Russia’s banks VTB and Pulkovo airport in St. Petersburg (the main transport hub in the North-West of the European part of Russia). The UAE and Saudi Arabia are more focused on investments in local infrastructural projects that have regional rather than federal importance. Thus, in the recent years Ayar International Investment Company participated in the reconstruction of St. Petersburg tram lines. The UAE money were invested in the development of IT software for Russian oncology and maternity centers. Mubadala contributed to the development of medical clinics in Podolsk and Balashikha. It also funded the construction of logistic complexes of Novosibirsk and Moscow districts. Meanwhile, the PIF participated in the reconstruction of petrochemical factory ZapSibNeftekhim in Tobolsk, construction of hydropower plant in the Karelian district and transport infrastructure of St. Petersburg (Shpilevskaya 2019). As of the early 2020, DP World was considering the purchase a 49% stake in Vladivostok-based transport company Fesco (Russia Business Today 2020).

Qatari, Emirati and Saudi approaches to investment in the Russia’s economy have their own pros and cons. However, to see all of them, one should take into account that apart from economic gains (that, according to the market analysts are not high) these investments can bring to the GCC political dividends (Galaktionova et al. 2018). Thus, the Qatari strategy of investing in the large companies definitely help Doha to create its lobby of supporters at the very top of the Russian elite of the federal level. These investments are also immediately visible for the Russian central authorities that also helps the Qatari government to gain additional scores in the eyes of Moscow. The efforts of the UAE and Saudi Arabia might, at the first approach, seem less important both economically and politically as the size of their investments in a single project might be counted less than in hundred thousands dollars with the low level of return while the project itself can be implemented somewhere far from the central cities of the country. However, this perception about the low effectiveness of such investments is deceiving. In the long run, the aggregated positive effect for the Russian economic development from the implementation of such projects might appear more viable than from the purchase of shares in Russia’s giants while also creating more deep-rooted political ties between the UAE, Saudi Arabia and Russia and opening options for participation in bigger and more profitable projects.

Russian business is also interested in entering the GCC. There is a distinct interest for Russian companies to enter the agro-industrial and nuclear sectors of the GCC economies, as well as create joint ventures in the field of telecommunications, IT-technologies as well as the mining and petrochemical sectors. As such, Russian economic interests are also becoming more diverse. The RDIF, for example, has actively lobbied the Russian national railway company to bid for railroad construction tenders in Saudi Arabia.

Moscow also views long-term economic projects as a solid base for the further development of bilateral ties with the region. Russia’s activities in the nuclear sector are an example of politically driven economic steps. Nuclear projects require long-term post-construction service contracts and would bind the countries concerned to Russia. In the case of the GCC countries, such ties could help ensure long-term economic cooperation in the absence of progress in other areas.

Russia’s relations with the GCC countries in the oil and gas field are not that straightforward. In the spring of 2020, the short-lived price war between Russia and Saudi Arabia demonstrated that, in spite of the deep interest in developing cooperation with Middle Eastern hydrocarbon producers put forward by Russia over the last four years, the alliances it has built remain fragile. Even positive bilateral dialogues with Middle Eastern exporters cannot offset challenges to Russia’s position in the global energy markets. The Kremlin is particularly worried about competition over the EU market.

It is often ignored by analysts that Russia and Saudi Arabia have periodically competed for oil markets in Asia and Europe in recent years. In 2018–2019, in spite of domestic production cuts, both increased their supplies to China in a competition for the spare share of the country’s market that had been created by a decrease in Iran’s oil exports to China, growing domestic demand, and Beijing’s attempts to diversify its sources of hydrocarbon imports. In the first half of 2019, Russia became the largest oil exporter to China, but by the beginning of 2020 Saudi Arabia had taken over this position. Saudi Arabia has also been a competitor to Russia in other regions. Its decision in July 2019 to further discount oil sold to Europe caused concern in Russia. These concerns strengthened again when following the Russian March 2020 decision to leave the OPEC + arrangement, Saudi Arabia declared its intention to provide European consumers with historically high discounts on its oil for April loaded cargoes. Moscow, however, equally never missed an opportunity to exploit the misfortunes of the Arab “partners”: in September 2019 following the attack by Iranian proxies on the Saudi oil-refining infrastructure in Abqaiq and Khurais led to Riyadh being temporarily unable to fulfil its export obligation to Asian countries. Russia immediately used this opportunity to position itself as a more reliable supplier to India in order to increase its share of the country’s market.

Yet, in most cases, Moscow still prefers cooperation over open confrontation. First of all Russia wants GCC investors to participate in joint ventures to research, design and produce oil, gas and petrochemicals equipment, given that current Western sanctions limit Moscow’s ability to import advanced Western technology. Russia pays special attention in this regard to cooperation with Saudi Arabia and the United Arab Emirates. In 2018, Saudi Aramco established a cooperation with Moscow University’s research centre on the development of the upstream technologies. Since 2017, the petrochemical company Sibur has been discussing options for entering the project for the construction of Al Jubail petrochemical factory, conducted by Saudi Aramco and Total. Saudi Aramco is also involved in negotiations with Rosneft and Lukoil over joint ventures in the petrochemicals sector. In 2018, the minister of energy, industry and mineral resources said that Saudi Aramco was also ready to invest in the efforts of Rosneft and Lukoil to buy or build refineries in third countries.

Second, the dependence of the Russian state budget on the exports of hydrocarbons and the Kremlin’s concerns about long-term low oil price compels Russia to cooperate actively with the Organization of the Petroleum Exporting Countries (OPEC), and in particular with Saudi Arabia. Russia’s decision to begin coordination of its output with OPEC producers can be named as historical. Until the mid-2010s, Moscow vision of its relations with the cartel was based on the principle of a free rider: while profiting from the OPEC attempts to regulate the market prices through the readjustment of oil during 1990s–2000s, Russia showed no interests in coordination with this structure. The OPEC members, in their turn, never insisted on such cooperation (Pravosudov 2020). Nevertheless, by the mid-2010s, the new trends at the global oil market made compelled Russia and OPEC to revise relations in order to protect their interests at the hydrocarbon market.

In 2016, joint Russian-Saudi efforts led to the Vienna Agreement between OPEC and non-OPEC countries (so-called OPEC +) to decrease production in order to ensure a degree of stability as far as oil prices were concerned. The initial six-month OPEC + deal has since been extended several times. It also led to the formation of a permanent forum-like structure with its own charter (signed in July 2019), which allows participants to coordinate and adjust their production policies. From Russia’s perspective, the arrangement proved beneficial as the oil price remained fairly high and stable at least temporarily. Thus, according to the Russian minister of energy, Aleksandr Novak, in 2019, the Vienna Agreement allowed Russia’s budget to accumulate about $32 bln (InvestFuture 2019). In December 2019, the deal was extended until April 2020. The announcement by Russia’s Energy Minister Aleksandr Novak on March 6, 2020, to withdraw Russia from the Vienna Agreement after April 1, 2020 however, revealed the fragility of the relationship. Moscow’s decision to stop the engagement was caused by the declining ability of OPEC + to affect the global oil market. By March 2020, Russia accepted that the era of high oil prices had an assessment clearly reflected in the Russian state budget planning that is built on the assumption of prices floating in the corridor $50–60 pb (likely closer to the lower end) until 2036 (Ministry of Finance of Russia 2019). Moscow also was convinced that oil prices would drop below $50 pb over the coming four years before returning to the $50–60 pb corridor. Russia’s leadership further sensed the growing influence of non-OPEC + members on oil prices as well as of forthcoming structural changes in market fundamentals that neither Russia alone nor OPEC + can control. At the same time, the Kremlin decided out of necessity to decrease the state budget’s dependency on oil in turn reflecting a degree of pessimism about the ability to maintain current oil output. Expected low prices, high upfront costs for the majority of new oil fields, and the lack of technologies and funds make one-third of Russian oil reserves unprofitable. Under these circumstances, the share of budget revenue from oil was expected to fall making Russia’s engagement with OPEC + less important for its economy.

However, Moscow appears to have underestimated the potential consequences of its withdrawal from OPEC + . The Kremlin either expected that its move would scare other participants to accept Russia’s demands not to deepen production cuts or assumed that the negative impact of the collapse of the existing arrangement would ultimately not be that dramatic. Instead, Russia was overtaken by events and quickly found itself in a full-fledged oil war. As the COVID-19 implications intensified and GCC countries, foremost Saudi Arabia, decided to expand oil production in an effort to gain market share, oil prices soon found themselves well below the $40pb threshold with no sense of any immediate recovery This scared the Kremlin and resulted in Minister Novak calling OPEC + members to keep their oil output within the limits observed in January–February 2020 less than a week after the initial announcement of the Russia withdrawal from the consortium (RNS 2020). The pressure on world energy markets ultimately resulted in a new production being renegotiated in April 2020.

In general, Russia is still defining its strategy on how to deal with the consequences for the shale revolution and the beginning of the global energy transition for the oil and gas markets. Alternatives consist of either losing a share of the oil market but sustaining high oil prices by limiting output with other members of the OPEC + agreement or to fight for market share at the expense of low oil prices. None of these options is ideal. It is important to keep in mind that Moscow participation in the OPEC + was restored by external circumstances as the Kremlin could not foresee the depth of the negative impact of the COVID-19 on global oil demand. Yet, this also means that, following a stabilization of oil markets, Russian oil producers could try to leave the OPEC + again.

## Conclusions

All in all, Russia should be considered a tier-2 non-regional player for the GCC states with a wide dialogue agenda but limited capacities to both pursue its interests and challenge either the interests of the GCC states or Western interests in the Gulf. Moscow is weak economically, which, in turn, weakens its political leverages of influence. In this case, the economic component became dominant and expanded along with an improvement in foreign economic relations. The construction of nuclear power plants, contracts in the energy and military-technical sphere and the launching of satellites show that Russia is a potential partner for the countries in the region.

Country-wise Russia’s relations with the GCC are uneven. Thus, Moscow’s trade and economic relations are more stable and better off with the UAE than the other countries. In terms of investments, Qatar remains a leader by volumes (yet, not by the quantity of investments) and hope to increase them in 2021 when Doha will be the main guest of the St. Petersburg International Economic Forum, a key economic event in Russia. The Russian-Saudi political dialogue remains one of the most productive when compared to Moscow’s relations with other players. However, on a broader scale, the GCC member states are facing one and the same issue: in order to move their relations with Russia further they need to overcome obstacles mentioned above.

While, in the near future, the qualitative evolution of the Russia’s relations with the Gulf is hardly possible, there are still options for their deepening. Russia’s strategy of constant balancing between key regional players, its limited political and economic resources as well as complexed relations of the GCC members states with the US, Iran and Turkey, the formation of long-lasting alliances/cooperation with Moscow on regional agenda will hardly be possible.

Nevertheless, Russia does not intend to withdraw from the Gulf as the region still has political and economic value for Moscow. Russia’s presence in the Middle East seeks to advertise its capacity to project power and helps Moscow avoid international isolation as well as weaken anti-Russian coalitions. Despite all its limitations, Russia can still play certain niche political and economic roles in the region. As this was discussed in the article, Moscow can build up ad hoc alliances with some Gulf states in Libya, help the Gulf players to secure their presence in Syria and be silently supportive of the Saudi coalition measures in Yemen.

The change of administration in the USA and the already emerging more diversified approach of Joseph Biden to the Middle East settlement also opens a certain window of opportunity for Moscow (TASS 2021). In January 2021, the Russian leadership made an attempt to take the lead in the Arab–Israeli conflict, immediately responding to the initiative of Palestinian Authority President Mahmoud Abbas to convene an international conference on a Middle East settlement. Moreover, according to Moscow, not only the countries of the Quartet on the Middle East, but also Egypt, Jordan, as well as some Gulf countries (Saudi Arabia, Bahrain and the United Arab Emirates) should take part in the preparation for this event.

The Russian capacities as a mediator that can deliver messages between all Middle Eastern players should not also be ignored when it comes to the GCC member states relations with Iran or between each other. Yet, to become an effective mediator, Russia will need not only to show the regional players that it is indifferent to Iranian interests (in other words, to ruin the strong belief existing among the GCC member states that it is a loyal protector of Iran), but to show that it can guarantee the fulfillment of any agreements reached during its mediation. In the last case, the Gulf countries are seriously doubting that Moscow can play the role of a guarantor.

Finally, in spite of potentially declining oil output Moscow will still remain one of the important players in the hydrocarbon markets facing the same set of problems as the crude producers of the Gulf (such as the impact of shale oil revolution on the markets and the beginning of energy transition to non-carbon fuels) and looking for ways to handle them through cooperation with the GCC.

Russia’s stable interest in developing relations with the GCC occasionally motivates the Kremlin to punch far above its weight. It can be argued that Russia will try to underline its importance and relevance by putting forward diplomatic initiatives that do not require much material investment that would expose Moscow’s limitations. Examples include capitalizing on topic such as the Syrian crisis, Libya and Israeli-Palestinian tensions in order to drag regional players into discussion of these issues.
